# Parental and Children’s Preference of Full-Coverage Restorations on Primary Molars: A Cross-Sectional Study

**DOI:** 10.3390/children13010081

**Published:** 2026-01-05

**Authors:** Sara M. Bagher, Hanouf J. Alharbi, Shahad N. Abudawood, Osama M. Felemban, Rahaf Sahhaf, Hanan Alagl

**Affiliations:** 1 Pediatric Dentistry Department, Faculty of Dentistry, King Abdulaziz University, P.O. Box 80200, Jeddah 21589, Saudi Arabia; 2 Ministry of Health, Aljouf Region, P.O. Box 24267, Makkah 74631, Saudi Arabia

**Keywords:** BioFlx, full-coverage restoration, preference, primary molars, stainless steel crown, zirconia

## Abstract

**Aim**: This cross-sectional study aimed to evaluate and compare parents’ and children’s preferences for full-coverage restorative treatment options of primary molars, including stainless steel crowns (SSCs), zirconia crowns (ZCs), and BioFlx crowns. Additionally, the study evaluates the influence of providing a brief overview of the advantages and disadvantages of each full-coverage restorative treatment option on parental preference. **Methods**: The study was conducted at the pediatric dental clinics at King Abdulaziz University Faculty of Dentistry (KAUFD) in Jeddah, Saudi Arabia, from January to May 2024. Healthy Arabic-speaking children aged 6–12 years attending KAUFD for routine dental treatment, along with at least one parent who agreed to participate, were included. Three typodont models with a SSC, a ZC, and a BioFlx crown were prepared and cemented by an expert pediatric dentist. The participating children and their parents were simultaneously and independently shown the prepared typodont models and asked to indicate which treatment option they preferred most. Subsequently, a trained pediatric dentist presented a brief overview of the advantages and disadvantages of each treatment option to the parents. Then, parents were asked to re-evaluate their preferences. The threshold for significance was set at *p* < 0.05. **Results**: A total of 172 children and their parents were included. The most preferred full-coverage restorative treatment among children was SSC (39.0%), while among parents, ZC (60.5%) was the most preferred. After providing a brief overview, the most preferred option among parents was SSC (39.5%), with ZC and BioFlx crowns being equally preferred (30.2%). Significantly more children with no history of dental pain or discomfort (49.1%) (*p* = 0.023) or with a history of previous dental treatment involving SSC (40.2%) (*p* = 0.045) preferred SSC. The ZC was significantly more preferred by parents of female children (70.65%) (*p* = 0.027) and by parents of children with a history of dental treatment (60.6%) (*p* = 0.018). **Conclusions**: The study revealed that parental demands and expectations often differ from those of their children, leading to notable differences between children’s and parents’ preferences. After a brief overview, parental preference shifted from ZC to SSC, highlighting the importance of effective communication and education when making treatment decisions for pediatric patients.

## 1. Introduction

Various restorative materials have been introduced in pediatric dentistry to preserve primary teeth before their permanent successors erupt [1]. One of the most durable, retentive, and affordable restorative materials is stainless steel crowns (SSCs). Stainless steel crowns have demonstrated good retention and clinical success in restoring larger carious lesions on primary molars [1]. Unfortunately, parents sometimes reject SSCs for aesthetic reasons [2]. Therefore, full-coverage aesthetic crowns, such as pre-veneered SSCs and prefabricated zirconia crowns (ZCs), have been introduced as alternative restorative treatment options [3,4,5,6].

Despite their superior aesthetic properties, ZCs also demonstrate excellent biocompatibility [5] and have been reported to be similar to SCCs in terms of crack and fracture incidence, along with lower gingival and plaque indices [2]. However, ZCs require more substantial reduction in tooth structure, offer limited crimping flexibility, and relatively high cost, which is becoming a barrier to treating patients with ZCs [2,6].

The new “NuSmile BioFlx” crowns were introduced in May 2023 as a middle-ground treatment option for restoring primary molars. According to the manufacturer, these are made from a biocompatible, high-impact resin polymer, free of phenol A-glycidyl methacrylate (Bis-GMA). These crowns are designed to offer high strength, flexibility, durability, and adaptability, while providing an aesthetic, full-coverage for posterior primary molars. [7]. According to a recent review by Al-Haj Ali (2025) [8], few studies investigated the physical and mechanical properties of Bioflx primary crowns, and only six clinical studies have assessed their clinical performance over follow-up periods up to 12 months. Of these, two were case reports and a case series, three were randomized controlled trials that compared BioFlx crowns with the SSCs, and one study evaluated BioFlx performance relative to both ZCs and SSCs. Both BioFlx crowns and SSCs demonstrated high clinical success rates in restoring primary molars [7,8,9]. In two clinical studies, Bioflx crowns had higher patient and parent satisfaction scores concerning esthetics compared to SSCs [10,11].

Aesthetic features, cost, toxicity, and durability were among the significant factors influencing parental preference for restorative treatment for their children [12]. Many authors have investigated parents’ and or children’s acceptance and attitudes toward SSCs. While most children reported being satisfied with SSCs and showed positive attitudes, parental acceptance and satisfaction were notably lower [12,13,14]. When evaluating clinical success and children’s and parental satisfaction with SSCs and ZCs in primary molars, both groups were significantly more satisfied with the color of the ZCs [13].

Today, children and parents are more aware of social media’s influence and the aesthetic treatments available, with increased focus on patient-centered studies that prioritize involving patients in decision-making [15,16]. Understanding parents’ and children’s expectations aids in establishing effective communication and in formulating a patient-specific treatment plan. Additionally, parental education about all available treatment options is essential for making an educated treatment decision for their children.

No previous research has evaluated parental and children’s preferences for the newly introduced BioFlx crowns compared with SSCs and ZCs, and the influence of parental education on the advantages and disadvantages of each treatment option when making a treatment decision for the children. Therefore, this cross-sectional study aims to assess and compare children’s and parental preferences for full-coverage restorative treatment options, including SSCs, ZCs, and BioFlx crowns for primary molars. Additionally, the study evaluates the influence of providing a brief overview of the advantages and disadvantages of each full-coverage restorative treatment option on parental preference.

## 2. Materials and Methods

### 2.1. Study Design and Setting

This descriptive cross-sectional survey of a convenient sample was conducted at the paediatric dental clinics at King Abdulaziz University Faculty of Dentistry (KAUFD) in Jeddah, Saudi Arabia, between January and May 2024. Ethical approval was obtained from the Research Ethics Committee at the Faculty of Dentistry, King Abdulaziz University, Jeddah, Saudi Arabia (212-11-23).

The inclusion criteria were healthy Arabic-speaking children aged 6 to 12 years who attended KAUFD for regular dental treatment, and at least one of their parents. Children with mental or cognitive disabilities were excluded. Using the OpenEpi sample size calculator (https://www.openepi.com, accessed on 1 November 2025), it was estimated that at least 146 child–parent pairs were required to estimate the preference for the crowns, assuming a large population and a hypothesized outcome frequency of 33 ± 5% (assuming equal distribution of children and parental preference among the three types of crowns) with an 80% confidence level.

Three trained paediatric dentists approached the parents/guardians of eligible children and explained the study’s aims and goals. Parents/guardians who agreed to participate signed an Arabic consent form before participation. Additionally, a verbal assent was obtained from the children.

### 2.2. The Questionnaire

The researchers developed a questionnaire in Arabic. Content validity was assessed by a panel of five expert associate professors and faculty members from the Department of Pediatric Dentistry, who reviewed and edited each question to ensure relevance, clarity, simplicity, importance, and absence of ambiguity.

Finally, the questionnaire’s reliability was assessed using a test–retest method, with a representative sample of 10 children and their parents who completed the questionnaire twice, two weeks apart. The responses were compared to assess test–retest reliability using the weighted Kappa, which was 0.98, indicating high reliability.

The questionnaire was divided into three separate sections. The first section collected demographic information about the participating children and their parents, including age, gender, the child’s rank among siblings, parental age, parental education level, the average monthly family income, and the child’s dental history. The child’s parental education was categorized into two groups: elementary/high school education or less, and college/higher education. The classification of average monthly family income was based on the Saudi Arabian Central Statistics and Information, where <7000 Saudi Riyals (SR) per month is considered low income; 7000–10,000 SR is middle; and >10,000 SR is high [17]. The child’s dental history included information on previous dental pain or discomfort, previous dental treatment, and whether the child had received SSC before as a full-coverage restoration treatment option in the primary molars.

In the second segment, the participating parents were asked to rate the importance of different factors (color, durability, and cost) in selecting a restoration for their children’s primary molars. The importance level was categorized into three levels: important, neutral, and not important.

In the third segment, the participating children and their parents were independently shown all three prepared typodont models at once and then interviewed by a trained pediatric dentist resident, who asked them to indicate which of the three full-coverage restorative treatment options they preferred most. The three full-coverage restorative options for primary molars, including SSC (3M ESPE, St. Paul, MN, USA), ZC (NuSmile, Houston, TX, USA), and BioFlx crowns (NuSmile, Houston, TX, USA), were prepared and cemented on three typodont models (Frasaco GmbH, Tettnang, Germany) by an expert pediatric dentist consultant.

Subsequently, the trained interviewer presented a brief overview of the advantages and disadvantages of each full-coverage restorative treatment option to the participating parents. Following this brief overview, the parents were interviewed again by the same pediatric dentist resident and asked to re-evaluate their preference.

The brief overview focused on the color, durability, and cost. The advantages of the SSC crown included durability and being the least expensive, with the disadvantage of an unnatural metallic color. In contrast, the ZC’s advantage was its durability and natural color, but it had the disadvantage of being the most expensive. Finally, the BioFlx advantages included durability and white color, being cheaper than the ZC but more expensive than the SSC, with the disadvantages of having less evidence of durability than the others and having less natural color than the ZC. All steps took place at the same dental clinic to ensure standardized lighting (Supplementary Material S1).

### 2.3. Statistical Methods

Data were entered and analyzed using SPSS Statistical analyses version 22.0 (SPSS Inc., Chicago, IL, USA). Categorical demographic variables were reported both as percentages and frequencies, while means and standard deviations were calculated from continuous data. The Chi-square test or Fisher Exact test were used to examine the association between categorical variables, with a predetermined significance threshold of *p* < 0.05.

## 3. Results

A total of 172 children and their parents participated in the study. The demographic characteristics of the participating children are presented in Table 1.

Most of the children had a history of dental pain or discomfort (117, 68.0%) and had undergone previous dental treatment (142, 82.6%). The treatments varied from prophylaxis and topical fluoride application (93, 54.1%), extractions (91, 52.9%), restorations (89, 51.7%), SSC (69, 40.1%), and pulp therapy (30, 17.4%). Parents of approximately 101 (58.7%) of the children reported that their child exhibited cooperative behavior during the previous dental visit.

**Figure 1:**
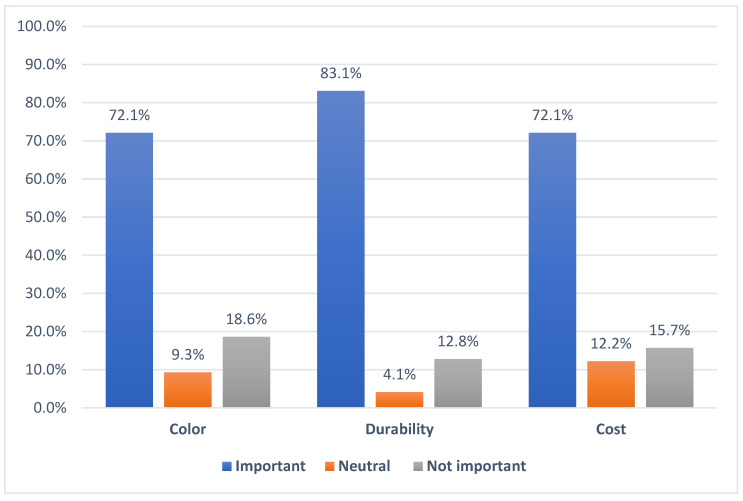
Distribution of the importance of color, durability, and cost among parents in selecting a restoration for their children’s primary molars.

When the parents were asked to rate the importance (important, neutral, not important) of different factors (color, durability, and cost) in selecting a restoration for their children’s primary molars, most reported that all three factors are very important, durability was chosen the most (143, 83.1%) followed by color (124, 72.1%) and cost (124, 72.1%). Figure 1 illustrates the distribution of the importance of color, durability, and cost among parents in selecting a restoration for their children’s primary molars.

**Figure 2:**
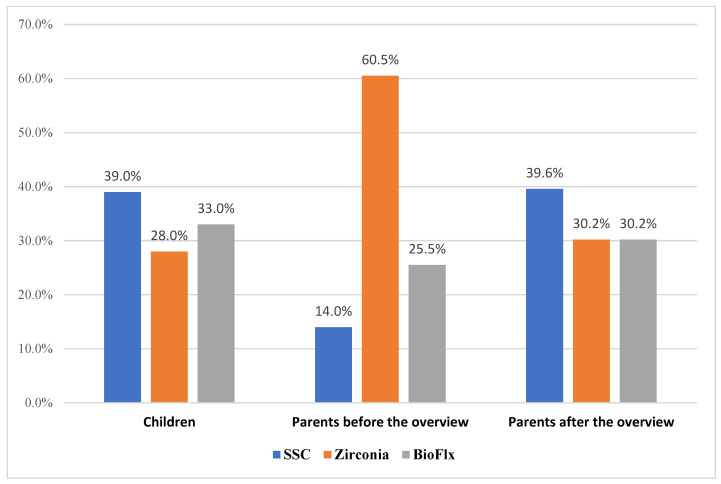
The most preferred full-coverage restorative treatment for primary molars among children and their parents.

Stainless-steel crown (67, 39.0%) was the most preferred among the children, followed by the BioFlx crown (57, 33.1%). Among the parents, the ZC (104, 60.5%) was initially the most preferred, followed by the BioFlx crown (44, 25.6%). Still, after the brief overview, their preference shifted toward SSC (68, 39.6%), with ZC and BioFlx crown being equally preferred (52, 30.2%). Figure 2 illustrates the most preferred full-coverage restorative treatment for primary molars among parents and their children.

Table 2 presents correlations between participants’ demographic characteristics and their preferred full-coverage restorative treatment before and after the brief overview.

Although most differences were not statistically significant, SSC was the most preferred among younger children (37, 42.5%), male children (36, 41.4%), children of parents with a college degree or higher (fathers (42, 40.8%), mothers (50, 42.4%)), and children from families with a middle average monthly income (23, 44.2%). In addition, children with a history of dental pain or discomfort (27, 49.1%) (*p* = 0.023) and those with no previous dental treatment involving SSC (39, 40.2%) (*p* = 0.045) preferred SSC.

Initially, ZC was significantly the most preferred by parents of female children (60, 70.6%) (*p =* 0.027) and by parents of children with a history of previous dental treatment (86, 60.6%) (*p =* 0.018). After the brief overview, although not significant, SSC became the most preferred among parents of older children (37, 43.5%), parents of female children (35, 41.2%), and ZC remained the most preferred among parents of families with a low average monthly income (20, 44.4%).

## 4. Discussion

The study aimed to assess and compare parents’ and children’s preferences for full-coverage restorative treatment options for primary molars, including SSC, ZC, and BioFlx crowns. It also evaluated how providing a brief overview of the advantages and disadvantages of each treatment to the parents affected their preferences. The results showed that SSC (67, 39.0%) was the most preferred option among children, while ZC (104, 60.5%) was the most preferred among parents. After the brief overview, the number of parents favoring SSCs and BioFlx crowns increased. Conversely, the number of parents who preferred ZC decreased, making SSC the most preferred treatment option among parents.

When the parents were asked to rate the relative importance of color, durability, and cost in selecting restorations for their children’s primary molars, all three were rated as important. These findings indicate that durability, financial considerations, and aesthetics are key determinants in parental decision-making. This was reflected in the initial parental preference for ZC. Once they were educated about the advantages and disadvantages of each full-coverage restorative treatment, more parents appeared to value the affordability of SSC, particularly given that all treatment options were described as durable in the brief overview provided. This finding agrees with a previous study by Utami et al. in 2020, who evaluated the attitude of children and their parents toward SSC and reported that most parents were worried about the metallic appearance and cost when compared to amalgam restorations [18].

The findings of the present study further emphasize that parental preferences, demands, and expectations often differ from those of their children, as children most preferred SSC. At the same time, ZC was the most favored option among parents. These results are consistent with previous studies that examined parental and children’s satisfaction with SSC and reported that children tend to have a more favorable perception of SSC than their parents [13,14,18]. This suggests that aesthetic and social considerations may weigh more heavily for parents than for children, who may prioritize novelty or personal appeal over appearance.

Before the brief overview, ZC was the most preferred by parents of female children, likely due to a stronger desire for aesthetic restorations for their daughters. Also, parents of children with a history of previous dental treatment significantly preferred ZC, and none of the parents of children with no prior dental treatment preferred SSC. This finding suggests that parents of children without prior dental experience may have lacked awareness of the advantages of SSC and, consequently, may have based their preferences predominantly on aesthetic considerations. After the overview, ZC remained the most preferred option among parents from low-income families (20, 44.4%). This suggests that parents from lower socioeconomic backgrounds may place greater value on the aesthetic appeal of restorative treatment options, possibly perceiving ZC to enhance their child’s appearance and social acceptance. However, it is essential to consider that the study was conducted at King Abdulaziz University, a governmental institution, where all treatment is provided free of charge. Therefore, the results may not accurately reflect the actual situation.

In the present study, the specific reasons underlying children’s and parents’ preferences before and after the brief overview were not investigated. However, it was previously reported that the natural tooth color of ZC was the primary reason for parental and children’s satisfaction with ZC. Additionally, some children reported that their negative perception of SSC was linked to social ridicule by peers [13,19]. However, the current study found that younger children, males, and those from middle-income families showed a higher preference for SSC. This may be attributed to the appeal of the metallic color, which younger and male children might find novel or entertaining, and to their potentially lesser susceptibility to media influence. However, it contrasts with findings from other studies, which suggested that younger children’s choices are more strongly influenced by their parents’ preferences compared to those of older children [20,21].

Further analysis revealed that most children who preferred SSC had no prior experience with it (39 out of 67; 58.2%). Similarly, most parents who preferred SSC for their children (15 out of 24; 62.5%) were also those whose children had not previously received SSC treatment. However, after the brief overview, the proportion of parents of children with prior SSC experience who preferred SSC increased to 32 out of 68 (47.1%), becoming comparable to the preference rate among those without prior SSC experience (36 out of 68, 52.9%). These findings indicate that parents with previous exposure to SSC on their children are initially less inclined to prefer it and more inclined toward the other, more appealing and natural-looking options. Nevertheless, once they became aware of the advantages and disadvantages of all treatment options, their appreciation for the SSC improved, reflecting a shift in perception from aesthetic concerns to durability and cost advantages.

In the current study, children and parents were shown a typodont model of the crowns and did not receive the treatment. Which explains the difference in the as the participants in the previously conducted In the current study, children and parents were shown a typodont model of the crowns and did not receive the treatment, similar to participants in previous clinical trials, in order to compare the clinical success and satisfaction of SSC and ZC in primary molars clinical trials and compared the clinical success and satisfaction of SSC and ZC in primary molars. In those trials, the preferences and satisfaction of the participating children and their parents were reported immediately or a few months (ranging from 6 to 36 months) after receiving the crowns [13,19,22].

In a 2020 study by Mathew et al. more children (100%) were satisfied with ZC than SSC (53.3%) [13]. Additionally, Bhatt et al. reported that only 27% of parents and 17% of children were satisfied with the metallic appearance of SSC, whereas 71% of parents and 90% of children were satisfied with the appearance of ZC [19]. When children and parents satisfaction with SSC was compared to composite restoration by Moslemi et al. 75% of the children who received SSC were satisfied compared to 85% of those who received composite restoration immediately after the treatment, but the rate of their satisfaction after one year decreased significantly to 69% among children who received SSC and increased to 90% among those who received composite restoration [22]. In contrast, Mathew et al. reported that the rate of children and parental satisfaction with both SSC and ZC did not change over the three-year study period [13].

Limitations of the study include a lack of longitudinal follow-up and its cross-sectional design with a limited number of convenience participants. However, it is essential to consider that the study was conducted at a single center, King Abdulaziz University, a governmental institution, where all treatment is provided free of charge. Therefore, the results may not accurately reflect the actual situation. Participants’ responses may have been influenced by the interviewer potentially introducing response bias. In addition, the use of 80% confidence level in our sample size calculation was intended to overcome the time constraints of completing the study; however, this may have negatively affected the precision of the study estimates. Also, although the interviewers were calibrated prior to the data collection, no formal measure of agreement was conducted.

Future investigations should aim to elucidate the impact of variables such as socioeconomic status, social media exposure, previous dental experience, children’s self-confidence, and peer influence on parental and children’s preferences and acceptance. These studies should be conducted on a larger scale and within clinical frameworks involving the actual placement of crowns.

## 5. Conclusions

The study revealed that parental demands and expectations differ from those of their children. The stainless-steel crown was the most preferred among children, while the zirconia crown was the most preferred among parents, particularly those of female children and those of children with a history of previous dental treatment. After the brief overview, parental preference shifted toward SSC, emphasizing the importance of effective communication and parental education when making treatment decisions for pediatric patients.

## Figures and Tables

**Table 1:** The demographic characteristics of the participating children and their parents (N = 172).

Variables		Number (%)
Child’s age in years	6–8	87 (50.6)
	9–12	85 (49.4)
Child’s gender	Male	87 (50.6)
	Female	85 (49.4)
Rank of the child among siblings	First	58 (33.7)
	Second	48 (27.9)
	Third or later	66 (38.4)
Fathers’ age	Mean ± SD	43.2 ± 7.3
Mothers’ age	Mean ± SD	37.9 ± 6.2
Father education	Elementary/high school education or less	69 (40.1)
	College/higher education	103 (59.9)
Mother education	Elementary/high school education or less	54 (31.4)
	College/higher education	118 (68.6)
Average monthly income of the family in Saudi Riyals	Low	45 (26.8)
	Middle	52 (30.2)
	High	53 (30.8)

**Table 2:** The correlations between participants’ demographic characteristics and their preferred full-coverage restorative treatment before and after the brief overview.

	Children’s Preferred Treatment Option N (%)			Parents’ Preferred Treatment Before the Brief Overview N (%)			Parents’ Preferred Treatment After the Brief Overview N (%)		
	SSC	ZC	BioFlx	SSC	ZC	BioFlx	SSC	ZC	BioFlx
Age in years									
6–8	37 (42.5)	18 (20.7)	32 (36.8)	10 (11.5)	54 (62.1)	23 (26.4)	31 (35.6)	29 (33.3)	27 (31.0)
9–12	30 (35.3)	30 (35.3)	25 (29.4)	14 (16.5)	50 (58.8)	21 (24.7)	37 (43.5)	23 (27.1)	25 (29.4)
*p*-value	0.102 €			0.641 €			0.528 €		
Gender									
Male	36 (41.4)	22 (25.3)	29 (33.3)	15 (17.2)	44 (50.6)	28 (32.2)	33 (37.9)	23 (26.4)	31 (35.6)
Female	31 (36.5)	26 (30.6)	28 (32.9)	9 (10.6)	60 (70.6)	16 (18.8)	35 (41.2)	29 (34.1)	21 (24.7)
*p*-value	0.704 €			0.027 * €			0.266 €		
Rank of the child among siblings									
First	25 (43.1)	12 (20.7)	21 (36.2)	6 (10.3)	35 (60.3)	17 (29.3)	22 (37.9)	18 (31.0)	18 (31.0)
Second	15 (31.3)	16 (33.3)	17 (35.4)	8 (16.7)	30 (62.5)	10 (20.8)	19 (39.6)	17 (35.4)	12 (25.0)
Third or later	17 (25.8)	20 (30.3)	29 (43.9)	10 (15.2)	39 (59.1)	17 (25.8)	27 (40.9)	17 (25.8)	22 (33.3)
*p*-value	0.259 €			0.799 €			0.808 €		
Father education									
Elementary/High school or less	25 (36.2)	16 (23.2)	28 (40.6)	6 (8.7)	45 (65.2)	18 (26.1)	23 (33.3)	24 (34.8)	22 (31.9)
College/higher	42 (40.8)	32 (31.1)	29 (28.2)	18 (17.5)	59 (57.3)	26 (25.2)	45 (43.7)	28 (27.2)	30 (29.1)
*p*-value	0.216 €			0.256 €			0.365 €		
Mother education									
Elementary/High school or less	17 (31.5)	15 (27.8)	22 (40.7)	4 (7.4)	34 (63.0)	16 (29.6)	23 (42.6)	16 (29.6)	15 (27.8)
College/higher	50 (42.4)	33 (28.0)	35 (29.7)	20 (16.9)	70 (59.3)	28 (23.7)	45 (38.1)	36 (30.5)	37 (31.4)
*p*-value	0.286 €			0.222 F			0.838 €		
Who is answering the Questionnaire?									
Father	21 (42.9)	13 (26.5)	15 (30.6)	9 (18.4)	31 (63.3)	9 (18.4)	16 (32.7)	13 (26.5)	20 (40.8)
Mother	46 (37.4)	35 (28.5)	42 (34.1)	35 (28.5)	73 (59.3)	15 (12.2)	36 (29.3)	39 (31.7)	48 (39.0)
*p*-value	0.800 €			0.295 €			0.790 €		
Average monthly income of the family in Saudi Riyals									
Low	18 (40.0)	12 (26.7)	15 (33.3)	2 (4.4)	30 (66.7)	13 (28.9)	15 (33.3)	20 (44.4)	10 (22.2)
Middle	23 (44.2)	13 (25.0)	16 (30.8)	7 (13.5)	33 (63.5)	12 (23.1)	20 (38.5)	14 (26.9)	18 (34.6)
High	26 (34.7)	23 (30.7)	26 (34.7)	15 (20.0)	41 (54.7)	19 (25.3)	33 (44.0)	18 (24.0)	24 (32.0)
*p*-value	0.867 €			0.282 F			0.174 €		
History of dental pain or discomfort									
No	27 (49.1)	8 (14.5)	20 (36.4)	4 (7.3)	32 (58.2)	19 (34.5)	21 (38.2)	18 (32.7)	16 (29.1)
Yes	40 (34.2)	40 (34.2)	37 (31.6)	20 (17.1)	72 (61.5)	25 (21.4)	47 (40.2)	34 (29.1)	36 (30.8)
*p*-value	0.023 * €			0.083 F			0.887 €		
History of previous dental treatment									
No	17 (56.7)	5 (16.7)	8 (26.7)	0	18 (60.0)	12 (40.0)	8 (26.7)	11 (36.7)	11 (36.7)
Yes	50 (35.2)	43 (30.3)	49 (34.5)	24 (16.9)	86 (60.6)	32 (22.5)	60 (42.3)	41 (28.9)	41 (28.9)
*p*-value	0.081 €			0.009 * F			0.284 €		
Previous dental treatment involving SSC									
No	39 (40.2)	20 (20.6)	38 (39.2)	15 (15.5)	55 (56.7)	27 (27.8)	36 (37.1)	32 (33.0)	29 (29.9)
Yes	28 (37.8)	27 (36.5)	19 (25.7)	9 (12.2)	48 (64.9)	17 (23.0)	32 (43.2)	19 (25.7)	23 (31.1)
*p*-value	0.045 * €			0.555 €			0.557 €		

* Statistically significant; € chi-square test; F Fisher Exact test.

## Data Availability

The Dataset is available on request from the authors. The data are not publicly available confidentiality considerations.

## Supplementary Materials

The following supporting information can be downloaded at: https://www.mdpi.com/article/10.3390/children13010081/s1, S1: brief explanation of the structured script.

## Abbreviations

The following abbreviations are used in this manuscript:

SSC	Stainless Steel Crown
ZC	Zirconia Crown
KAUFD	King Abdulaziz University Faculty of Dentistry
SR	Saudi Riyals
