# Supplementary material for: Parental and Children’s Preference of Full-Coverage Restorations on Primary Molars: A Cross-Sectional Study
Source: Children (Basel). 2026 Jan 5;13(1):81. doi: 10.3390/children13010081 (PMC12840054; doi:10.3390/children13010081)
Supplement: Supplementary file 1 [file children-13-00081-s001.zip › children-4018059-supplementary.pdf]

Methodology brief explanation of the three crowns (supplementary material S1)

A structured script was used by a trained interviewer to discuss the advantages and the disadvantages of each full-coverage restorative treatment option to the participating parents focusing on the color, durability and cost. It was mentioned in the methodology section that the advantages of the SSC crown included durability and being the least expensive, with the disadvantage of an unnatural metallic color. In contrast, the ZC's advantage was its durability and natural color, but it had the disadvantage of being the most expensive. Finally, the BioFlx advantages included durability and white color, being cheaper than the ZC but more expensive than the SSC, with the disadvantages of having less evidence of durability than the others and having less natural color than the ZC
